# *Bacillus subtilis* Smc condenses chromosomes in a heterologous cell system, which is down-regulated by ScpAB

**DOI:** 10.1186/s13104-020-05344-3

**Published:** 2020-11-11

**Authors:** Tobias Knust, Peter L. Graumann

**Affiliations:** 1 Agilent Technologies R&D and Marketing GmbH & Co. KG, Hewlett-Packard-Strasse 8, 76337 Waldbronn, Germany; 2 SYNMIKRO, LOEWE-Zentrum Für Synthetische Mikrobiologie, Hans-Meerwein-Straße, 35043 Marburg, Germany; 3 Fachbereich Chemie, Hans-Meerwein-Straße 4, 35032 Marburg, Germany

**Keywords:** Structural maintenance of chromosomes, SMC, *Bacillus subtilis*, Chromosome condensation

## Abstract

**Objective:**

Structural maintenance of chromosomes (SMC) proteins are key players in chromosome dynamics in all types of organisms. The so-called condensin subfamily is essential for chromosome condensation in eukaryotic cells, as is the bacterial SMC complex (called MukBEF in *Escherichia coli*). We expressed the *Bacillus subtilis* Smc protein and its two complex partners ScpA and ScpB in *E. coli* cells, and monitored effects on chromosome compaction by DNA staining of live cells using epifluorescence microscopy.

**Data description:**

We show that expression of BsSmc leads to strong chromosome compaction, while expression of ScpAB does not show any effect. Chromosome compaction by Smc was also found for mutant versions lacking ATP binding or ability for head engagement, and was counteracted by concomitant expression of ScpAB. Our findings show that the SMC complex can act as autonomous condensation system in a heterologous bacterial host system, for which neither ATP binding nor ATP hydrolysis are required. Our investigation suggests that the negative effect on compaction activity of Smc exerted by ScpAB in vivo does not involve an effect on ATPase activity, but more likely a stabilization of the engagement of head domains, which in turn may affect ATPase activity.

## Objective

Deletion of *smc*, *scpA* or *scpB* genes in many bacteria, including *B. subtilis*, leads to slow, temperature sensitive growth, a defect in chromosome condensation/nucleoid compaction, and the generation of about 15% of cells lacking any chromosome (anucleate cells) [1–4]. Smc, ScpA and ScpB form a complex in vivo and in vitro [5, 6], and ScpA and ScpB form a sub-complex [7, 8] that affects ATPase activity of Smc [9]. Higher levels of Smc in *B. subtilis* cells result in chromosome hyper-compaction [7]. We wished to investigate if Smc and/or its complex partners ScpA and ScpB, which do not bind to DNA by themselves [10], can also condense chromosomes when expressed in other bacterial species. For this purpose, we chose *E. coli* cells as a model system, and employed a two-plasmids expression system. The PCR products of *scpA* and of *scpB* were cloned in tandem into pETDuet-1 (Novagen). *Smc* was cloned into pCDFDuet-1 (Novagen). Mutants of *smc* were created by the QickChange-protocoll. 20 ml culture of Rosetta 2 (DE3) pLysS cells hosting the desired vector was inoculated to an OD_600_ of 0.05 in LB containing required antibiotics and incubated on a shaking platform at 37 °C until OD_600_ 0.9 was reached. Expression was induced with 0.5 mM IPTG for Smc or Smc mutants, or with 3 mM IPTG for ScpAB expression. We monitored expression via SDS-PAGE analyses (Fig. 1), and investigated changes in nucleoid morphology using epifluorescence microscopy and 20 µg/ml of DAPI to stain DNA, and 5 µg/ml of FM4-64 to stain membranes.

## Data description

We set up low-level expression of Smc and/or high level for ScpAB in *Escherichia coli* cells, which have the highly diverged MukBEF complex instead of SMC/ScpAB [11]. Smc and mutant alleles were expressed for 60 min at similar levels (Fig. 1), and experiments were performed at least 3 times. Large *E. coli* cells cells (> 4 µm) contained a single bilobed nucleoid or two separated nucleoids, while small (< 3 µm) and middle-sized (3 to 4 µm) contained a single nucleoid (Fig. 2A). When ScpAB were expressed in the cells, no detectable change in nucleoid morphology could be detected (Fig. 2B). ScpAB were expressed at higher level than Smc or mutant Smc, also when expressed by themselves (Fig. 1), to rule out that effects generated by Smc are caused by simple protein overproduction. When wild type Smc was expressed in *E. coli* cells, chromosomes were markedly more compacted than in the absence of expression, or during expression of ScpAB (Fig. 2C, compare with 2A and 2B) with larger space between the nucleoid and the cell poles. Line scans through nucleoids in middle sized cells (2.9–4.2 µm) expressing Smc showed markedly less DNA in the cell centre (Fig. 2J) than in control cells (Fig. 2H) or in cells expressing ScpAB (Fig. 2I), revealing that more middle-sized cells contained two separated nucleoids than in the absence of Smc expression. Also, nucleoids were narrower during Smc expression, as seen by the different slope of the edges of the line scans (Fig. 2 J, compare with 2H). Thus, Smc can hypercondense chromosomes in a heterologous cell system. When ScpAB were co-expressed with Smc, chromosomes were less condensed than during expression of Smc alone (Fig. 2D and 2 K, compare with 2C and 2 J) (Fig. 2 J). These data suggest that in vivo, ScpAB down-regulate condensation activity of Smc in a heterologous cell system. Interestingly, overproduction of MukB in *E. coli* also leads to hypercondensation, but overexpression of MukB together with its two complex partners MukE and MukF leads to more pronounced compaction [12], indicating considerable differences between MukBEF and Smc/ScpAB systems.

Expression of ATP-binding and head-engagement Smc proteins led to hypercompaction of chromosomes, stronger than wild type Smc during the expression of ATP-binding mutant D1117A (Fig. 2E), or comparable to that of wild type Smc in case of head-engagement mutant S1090R (Fig. 2F), see corresponding line scans (Fig. 2L and 2 M). Co-expression of ScpAB with each mutant Smc counteracted the compaction activity (not shown). The line scan for mutant D1117A plus ScpAB (not shown) showed a profile similar to overexpression of Smc and ScpAB (Fig. 2K). These experiments show that ScpAB also affect condensation activity of ATP mutant Smc in vivo. According to current views, SMC proteins can extrude large DNA loops in an ATP dependent manner [13] and thereby compact chromosomes, and ScpA and ScpB lead to structural rearrangements between SMC head domains that directly affect ATP binding [9, 14].

A mutation next to the Walker B motif (E1118Q) deceases ATPase activity, and stimulates DNA binding in an ATP-dependent manner [6]. Expression of SmcE1118Q in *E. coli* cells led to a strong chromosome segregation defect, while cells were considerably elongated (Fig. 2G). Chromosomes showed a variety of shapes, from decondensed to hypercondensed, many were also stretched, and 19% of the cells showed a cut phenotype (chromosomes bisected by a division septum). In 25% of the cells the chromosome was unevenly located (Fig. 2G). Because of the uneven nucleoid localization, it did not make sense to generate line scans, but clearly, the E1118Q mutation blocks efficient chromosome segregation in *E. coli*, similar to its effect in *B. subtilis* [15], and has a very different phenotype than the other two ATP mutants.

## Limitations

The *B. subtilis* SMC complex was expressed in wild type *E. coli* cells containing the endogenous MukBEF complex. We cannot rule out that the BsSMC complex has an effect on the activity of the MukBEF complex. We used an *E. coli* strain optimized for protein expression, so it is possible that other *E. coli* strains behave differently from the Rosetta strain employed. Because ScpAB were highly overproduced, their effect on mutant Smc proteins may be different from a scenario with wild type protein copy numbers. We cannot address whether ATP binding or ADP and phosphate release may be affected by ScpAB.

## Data Availability

The data described in this Data note can be freely and openly accessed on ***[figshare]*** under https://doi.org/10.6084/m9.figshare.12681779.v3 (Fig. 1) [16] and https://doi.org/10.6084/m9.figshare.12681788.v4 (Fig. 2) [17]. Please see Table 1 for details and links to the data.

**Table 1:** Overview of data files/data sets

Label	Name of data file/data set	File types (file extension)	Data repository and identifier (DOI or accession number)
Data file 1	Figure 1	JPEG	figshare: https://doi.org/10.6084/m9.figshare.12681779.v3 [16]
Data file 2	Figure 2	TIF	figshare: https://doi.org/10.6084/m9.figshare.12681788.v4 [17] Overview of data files/data sets figshare: https://doi.org/10.6084/m9.figshare.12681788.v4 [17]

## Abbreviations

FM4-64: N-(3-triethylammoniumpropyl)-4-(6-(4-(diethylamino)phenyl)hexatrienyl) pyridinium dibromide
DAPI: 4′,6-Diamidin-2-phenylindol (DAPI
